# Identifying the *BRCA1* c.-107A > T variant in Dutch patients with a tumor *BRCA1* promoter hypermethylation

**DOI:** 10.1007/s10689-022-00314-z

**Published:** 2022-09-16

**Authors:** Vincent M. T. de Jong, Roelof Pruntel, Tessa G. Steenbruggen, Fonnet E. Bleeker, Petra Nederlof, Frans B. L. Hogervorst, Sabine C. linn

**Affiliations:** 1 Department of Molecular Pathology, Netherlands Cancer Institute, Plesmanlaan 121, 1066CX Amsterdam, Netherlands; 2 Department of Pathology, The Netherlands Cancer Institute, Amsterdam, Netherlands; 3 Department of Medical Oncology, The Netherlands Cancer Institute, Amsterdam, Netherlands; 4 Department of Clinical Genetics, The Netherlands Cancer Institute, Amsterdam, Netherlands; 5 Department of Pathology, University Medical Center Utrecht, Utrecht, Netherlands

**Keywords:** BRCA1, Methylation, Breast cancer

## Abstract

An inherited single nucleotide variant (SNV) in the 5′UTR of the *BRCA1* gene c.-107A > T was identified to be related to *BRCA1* promoter hypermethylation and a hereditary breast and ovarian cancer phenotype in two UK families. We investigated whether this *BRCA1* variant was also present in a Dutch cohort of breast and ovarian cancer patients with tumor *BRCA1* promoter hypermethylation. We selected all breast and ovarian cancer cases that tested positive for tumor *BRCA1* promoter hypermethylation at the Netherlands Cancer Institute and Sanger sequenced the specific mutation in the tumor DNA. In total, we identified 193 tumors with *BRCA1* promoter hypermethylation in 178 unique patients. The wild-type allele was identified in 100% (193/193) of sequenced tumor samples. In a large cohort of 178 patients, none had tumors harboring the previously identified c.-107A > T SNV in *BRCA1*. We therefore can conclude that the germline SNV is not pervasive in patients with tumor *BRCA1* promoter hypermethylation.

## Introduction

Breast cancer is the most common type of cancer in women [1]. In some cancer patients, genetic predisposition plays a role, and in families with many breast cancer diagnoses, the possibility of an underlying genetic predisposition increases substantially. Pathogenic germline variants in the *BRCA1* and *BRCA2* genes are present in 3% of all breast cancer cases [2]. A recent estimation is that germline mutations in high-risk genes linked to breast cancer, including *BRCA1*, *BRCA2*, PALB2, *PTEN*, *TP53*, *CDH1*, and *STK11*, combined explain approximately 20% of the genetic predisposition [3, 4]. Parts of the missing predisposition have been attributed to polygenic variants and genes with moderate penetrance, including *CHEK2*, and *ATM* [3–6]. Next to pathogenic germline variants in genes, germline epigenetic silencing may also increase the risk of cancer. For instance, Lynch syndrome is known for its increased risk of cancer due to germline epigenetic silencing; a mechanism that might also be associated with other types of cancer [7]. In breast cancer, new strategies are being developed to detect heritable hypermethylation in families, however, so far without success [8, 9].

Of all sporadic breast tumors, it is estimated that 5–20% harbor hypermethylation of the *BRCA1* promoter, depending on the case mix studied [10–14]. *BRCA1* promoter hypermethylation is especially known to be associated with the triple-negative subtype, defined by its absence of expression of the estrogen, progesterone, and HER2 receptor [10, 15]. In 2018, Evans et al. identified an inherited 5′UTR single nucleotide variant (SNV) c.-107A > T linked to epigenetic silencing of the *BRCA1* gene. The epigenetic silencing of *BRCA1* was present in both germline and tumor DNA. Forty-nine patients from families with a high risk of developing breast or ovarian cancer (Manchester score of > 34) without a known germline pathogenic *BRCA1* mutation were examined. The Manchester score is based on family history and pathological characteristics of the tumor and indicative of the risk of a germline *BRCA1* or *BRCA2* mutation for patients with breast or ovarian cancer [16]. Two families were identified to carry the *BRCA1* c.-107A > T SNV and in these two families this variant was associated with an increased risk of breast and ovarian cancer [17].

In 2020, a study from South-East Germany failed to identify the germline presence of *BRCA1* c.-107A > T SNV in a large population, including 3297 patients with a high familial risk to develop breast and ovarian cancer, without a germline *BRCA1* or *BRCA2* mutation. These results indicated that the incidence of the *BRCA1* c.-107A > T SNV may be low [18]. For the Dutch population, the prevalence of the *BRCA1* c.-107A > T SNV is unknown. Information on the prevalence of this SNV could have implications for genetic counseling, screening, and prophylactic surgeries [19]. Therefore, we selected patients with a proven hypermethylated *BRCA1* promoter in their tumor to increase the chance of finding the SNV. In this study, we investigated the occurrence of the *BRCA1* c.-107A > T SNV in 178 patients, most of them with triple-negative breast cancer, who tested positive for *BRCA1* promoter hypermethylation by MLPA in their breast or ovarian tumor.

## Subjects and methods

### Patient selection

We selected all patients with breast or ovarian cancer with promoter hypermethylation of *BRCA1* in their tumor tested between 01-08-2007 and 01-09-2019 at the Netherlands Cancer Institute. Clinical information was obtained from the electronic health record of the institute. For the patients known in our clinical genetics department, the Manchester score was calculated by a clinical geneticist (FB) [16].

### Methylation assay

Methylation of the *BRCA1* promoter was previously determined by MS-MLPA (kit ME001 or kit ME005-custom) (MRC-Holland, Amsterdam). Analyzes and the cutoff were done according to manufacturer’s protocol. We used a ratio of 0.2 to define hypermethylation [11]. Earlier research showed that with the cutoff used here (0.2) very low *BRCA1* gene expression was found, pointing towards almost complete promotor hypermethylation [15, 20].

### PCR and sanger-sequencing

Tumor DNA was amplified and sequenced using BigDye™ Terminator v1.1 Cycle Sequencing Kit (Thermofisher, USA, Waltham), according to manufacturer’s protocol. To detect the NM_007294.4:c.-107A > T SNV (hg19) we used the following primers: Forward TTCTGAGAGGCTGCTGCTTA, Reverse AAACCCCACAGCCTGTCC. Sequences were analyzed using Mutation Surveyor (Softgenetics, Pennsylvania, USA).

## Results

We identified 193 tumor samples with *BRCA1* promoter hypermethylation in 178 unique patients (Table 1). For most patients the tumor promoter methylation status was tested in the context of a clinical trial: n = 50 Neo-TN (NCT01057069), n = 17 Triple-B (NCT01898117), OLIGO n = 5 (NCT01646034), and SUBITO n = 24 (NCT02810743). All other patients were tested during regular diagnostics or in a research setting. Of the identified patients, none (0%) had the *BRCA1* c.-107A > T SNV. For 51 patients familial information was available for review in our institute, all had tested negative for a germline pathogenic *BRCA1* or *BRCA2* variant. They had a median Manchester score of 9 (range 2–30).

**Table 1.** Patient and tumor characteristics

	N (%)
Median age, year (range)	40 (24–76)
Gender, no. (%)	
Female	178 (100%)
Median manchester score, (range)	9 (2–30)
Missing no. (%)	127 (72%)
Tumor origin, no. (%)	
Breast^a^	183 (95%)
Ovarian	10 (5%)

^a^134 tumors were triple-negative, 2 estrogen receptor-positive HER2 negative, and of 47 tumors information on receptor status was missing

## Discussion

In this study, we aimed to identify the prevalence of the *BRCA1* c.-107A > T SNV in breast or ovarian cancer patients with tumor *BRCA1* promoter hypermethylation. In our cohort of 178 patients with a tumor *BRCA1* promoter hypermethylation, we did not find any sample harboring the specific *BRCA1* c.-107A > T SNV. Our study design was based on the presence of *BRCA1* hypermethylation, rather than based on familial risk, in contrast to previous research [17, 18].

In the original study of Evans et al., the specific *BRCA1* c.-107A > T variant was identified in 2/49 high-risk families, all patients (n = 7) with *BRCA1* promoter hypermethylation harbored the same germline variant [17]. Notably, their selection included patients from families affected by breast and ovarian cancer without a germline *BRCA1* or *BRCA2* mutation, while our selection was focused on the presence of tumor *BRCA1* promoter hypermethylation. With the used methylation assay, tumor *BRCA1* promoter hypermethylation and a germline *BRCA1* mutation seem mutually exclusive [11, 21, 22]. In general it seems that co-occurrence of *BRCA1* promoter hypermethylation and a germline *BRCA1* mutation is extremely rare. Of the 51 patients known at our genetics department, not a single patient had a germline *BRCA1* or *BRCA2* mutation and none had the same familial risk as patients in the study by Evans and colleagues. Of note, for the majority of patients included in this study the familial risk is unknown. In contrast, the study of Laner et al. included all patients who had wild-type germline *BRCA1/2* and fulfilled the criteria necessary for genetic testing in Germany, which is less strict than the criteria used by Evans and colleagues [18]. Similar to our findings, Laner and colleagues did not detect the SNV in any of the 3297 patients tested. Furthermore, the two presumed unrelated families identified by Evans et al. shared a common ancestral haplotype, very indicative of a common ancestor [17].

Our study has a clear strength since it focused on a cohort of patients with a proven tumor *BRCA1* promoter hypermethylation. However, our study also has some limitations. Firstly, we did not have the Manchester Score for all patients limiting our ability to establish their familial risk. Nonetheless, the study from Laner et al. did not identify any patients with the SNV either even though they were selected for hereditary predisposition. Secondly, although the studied population is relatively large, this is not a representative selection of all breast cancer patients in The Netherlands. Therefore, we cannot definitively reject the hypothesis that the SNV plays a role in the familial breast or ovarian cancer risk for some Dutch patients. However, if the SNV was prevalent in the population, we would expect it to be included in the gnomAD database, which it is not [23]. Thirdly, we investigated tumor DNA for the SNV instead of germline DNA. We do not think investigating tumor DNA is an issue since all samples were identified as wild-type and a somatic reverse mutation in the tumor would be extremely unlikely. In case we had identified the SNV in any of the tumor samples, our next step would have been to test the germline DNA for this SNV.

To conclude, the *BRCA1* c.-107A > T SNV is not prevalent in a large cohort of patients with tumor *BRCA1* promoter hypermethylation. Given these results and those from previous studies, this germline variant does not seem to have a high prevalence in the Western-European population.

## Data Availability

All data generated or analyzed during this study are included in this published article.
